# Ice cage: new records and cryptic, isolated lineages in wingless snow flies (Diptera, Limoniidae: *Chionea* spp.) in German lower mountain ranges

**DOI:** 10.1007/s00114-024-01900-0

**Published:** 2024-03-13

**Authors:** Robert Klesser, Theo Blick, Michael-Andreas Fritze, Andreas Marten, Michael Hemauer, Laura Kastner, Hubert Höfer, Gero Jäger, Martin Husemann

**Affiliations:** 1 Leibniz Institut zur Analyse des Biodiversitätswandels, ztm, Zoologisches Museum Hamburg Martin-Luther-King-Platz 3, D-20146 Hamburg, Germany; 2 Naturkundemuseum Leipzig, Lortzingstraße 3, D-04105 Leipzig, Germany; 3 Private researcher, Heidloh 8, D-95503 Hummeltal, Germany; 4 Arbeitsgruppe für Tierökologie und Planung GmbH, Johann-Strauß-Str. 22, 70794 Filderstadt, Germany; 5 Harz National Park, Lindenallee 35, D-38855 Wernigerode, Germany; 6 Private researcher, Wallbergstraße 20, D-81539 Munich, Germany; 7 Staatliches Museum für Naturkunde Karlsruhe, Erbprinzenstr. 13, D-76133 Karlsruhe, Germany; 8 Private researcher, Fuldatalstraße 55, D-34125 Kassel, Germany

**Keywords:** Glacial relicts, Microclimate, Refuge, Stone run, Supranivean insects, Subnivean insects

## Abstract

In Earth’s history warm and cold periods have alternated. Especially, during the Pleistocene, the alternation between these different climatic conditions has led to frequent range expansions and retractions of many species: while thermophilic species dispersed during warm periods, cold adapted species retracted to cold refugia and vice versa. After the last Pleistocene cycle many cold adapted taxa found refuges in relict habitats in mountain ranges. One example for such a cold adapted relict is the flightless snow fly *Chionea araneoides* (Dalman, 1816). It can be found in lower mountain ranges of Central Europe exclusively in stone runs and stony accumulations which provide cold microclimates. Imagines develop only in winter. They have strongly restricted ranges and hence experienced strong isolation predicting that local populations may show local adaptation and hence also genetic differentiation. We investigated this for several middle mountain ranges of Germany using the COI barcoding gene. Our analyses revealed two distinct lineages, one in the Bavarian Forest and a second one in all other more northern locations up to Scandinavia. These lineages likely go back to post-Pleistocene isolation and should be studied in more detail in the future, also to confirm the taxonomic status of both lineages. Further, we confirmed former records of the species for Germany and report new records for the federal states of Saxony, Lower Saxony, Saxony-Anhalt and Thuringia. Finally, we provide the first evidence of two types of males for the species, a small and a larger male type.

## Introduction

Most terrestrial arthropods are rather thermophilic, leading to a steep diversity gradient from northern to southern latitudes and to main activity periods in summer; at least at northern latitudes. An exception are cold-adapted arthropod species, which are mainly active during winter in Central Europe (Soszynska 2004). A preference for cold temperatures evolved in several arthropod lineages as predator avoidance mechanism and is accompanied by a special physiology (Strathdee and Bale 1998). In order to survive temperatures below the freezing point, while being active, poikilothermic animals need physiological adaptations (Sömme and Östbye 1969). The main adaptation is the production of antifreeze agents, such as sugars, polyols and proteins (Duman et al. 2004; Vanin et al. 2008; Zachariassen 1985).

Stone runs represent a refuge habitat characterized by a very specific and cold microclimate (Molenda 1996). In Germany, outside the Alps, these specific habitats are largely restricted to the lower mountain ranges (Mittelgebirge). Their unique geomorphology induces some very special traits. Firstly, the rocky accumulations without any substrate between the rocks provide an extensive system of small and medium-scaled cavities under their surface (Molenda and Möseler 1999). Secondly, such cavity system in combination with a strong inclination allows for a natural air flow resulting from airstreams through the body of a stone run (Růžička and Zacharda 2010; Molenda 1996). In winter, warm air leaves the slope in in the upper areas, sucking in cold air at the base of the slope. In summer, this effect reverses. These alternating airstreams lead to cold air conditions in the lower parts of stone runs in summer and warm air in the higher parts in winter (Wakonigg 2017). Some authors have even suggested that permafrost may persist in the deep parts inside of some stone runs (Gude et al. 2003; Möseler and Wunder 1999). In the absence of higher vascular plants, lichens and mosses represent the main vegetation (Ott and Jahns 1999; Lüth 1999), often accompanied by barren rocky debris. This special composition of physico-chemical and biological traits makes the stone runs a periglacial microclimatic refuge, with a specialized fauna of cold-adapted arthropods (Molenda 1996; Růžička et al. 2012; Zacharda et al. 2005), e.g. *Wubanoides uralensis lithodytes* Schikorah, 2004, *Lepthyphantes notabilis* Kulczyński, 1887, *Bathyphantes eumenis buchari* Růžička, 1988 (Araneae), *Leistus piceus* Froelich, 1799, *Leistus montanus* Stephens, 1827, *Pterostichus negligens* Sturm, 1824, *Choleva lederiana lederiana* Reitter, 1902, and *Leptusa simoni* Eppelsheim, 1878 (Coleoptera) (Fritze and Blick 2010; Hadulla and Wagner 2016; Klesser et al. 2022). Some of these species are considered to be periglacial relicts, others represent alpine or montane specialists, which found habitat and climatic conditions similar to their typical habitats in higher mountain ranges in the stone runs.

One interesting cold adapted taxon are crane flies of the genus *Chionea* Dalman, 1816 within the family Limoniidae. All members of the genus are long legged and wingless Diptera and are mainly active in the winter period of the year. Some species are habitat specialists and occur in caves, stone runs and other rocky outcrops or in animal burrows (Oosterbroek and Reusch 2008) and several have restricted ranges, e.g. the endemic *C. pyrenaea* (Bourne, 1981) in the mountain ranges of the Pyrenees (D’Amico and Oosterbroek 2013). *Chionea* flies can live in Europe in altitudes up to 3000 m (Oosterbroek and Reusch 2008).

In Germany five species of *Chionea* are known: *Chionea araneoides* Dalman, 1816, *C. austriaca* Christian, 1980, *C. alpina* Bezii, 1908, *C. belgica* Becker, 1912 and *C. lutescens* Lundström, 1907. While *C. belgica* and *C. lutescens* have a wide distribution in Germany in all habitats and altitudes, *C. alpina* and *C. austriaca* are only known from the German Alps (Oosterbroek and Reusch 2008; Blick and Zaenker 2016). A very special case is *C. araneoides*, which is in Central Europe, outside of the high mountain systems, only recorded from and around rock structures, stony accumulations and block stone runs with a focus on the last habitat type (Blick and Fritze 2009). The species is widely distributed in Europe in higher mountain ranges (> 1000 m) and the boreal zone of Scandinavia. Below this altitude and outside the boreal zone it is only recorded from stone runs, stony debris and rocks. Records are known from the Alps (Austria, Italy, Romania, Switzerland), Northern Europe (Finland, Norway, Sweden), the Tatra (Slovakia) and several mountain ranges in Central and Eastern Europe (Czech Republic, Poland, Russia, Slovenia, Slovakia) (Oosterbroek and Reusch 2008). In northern Europe it seems to be more common without strong habitat preferences (Oosterbroek and Reusch 2008). In Germany it has been found so far in the Fichtel Mountains, in the Bavarian Forest (Bavaria) (Blick and Fritze 2009), the Rhoen Mountains (Thuringia and Hesse) (Bellstedt et al. 2014, Blick unpubl.), the Hoher Meißner in Hesse (Blick & Jäger, unpubl.) and in a cave in the German Alps (Blick and Zaenker 2016). In this study, we provide new records for the species from a larger trapping campaign including most German low mountain ranges (Mittelgebirge). We generated DNA barcodes for specimens from all sampling locations with the aim to understand the distribution and biogeography of the species and use the genetic data to test for cryptic diversity.

## Materials and methods

### Sampling

**Fig. 1:**
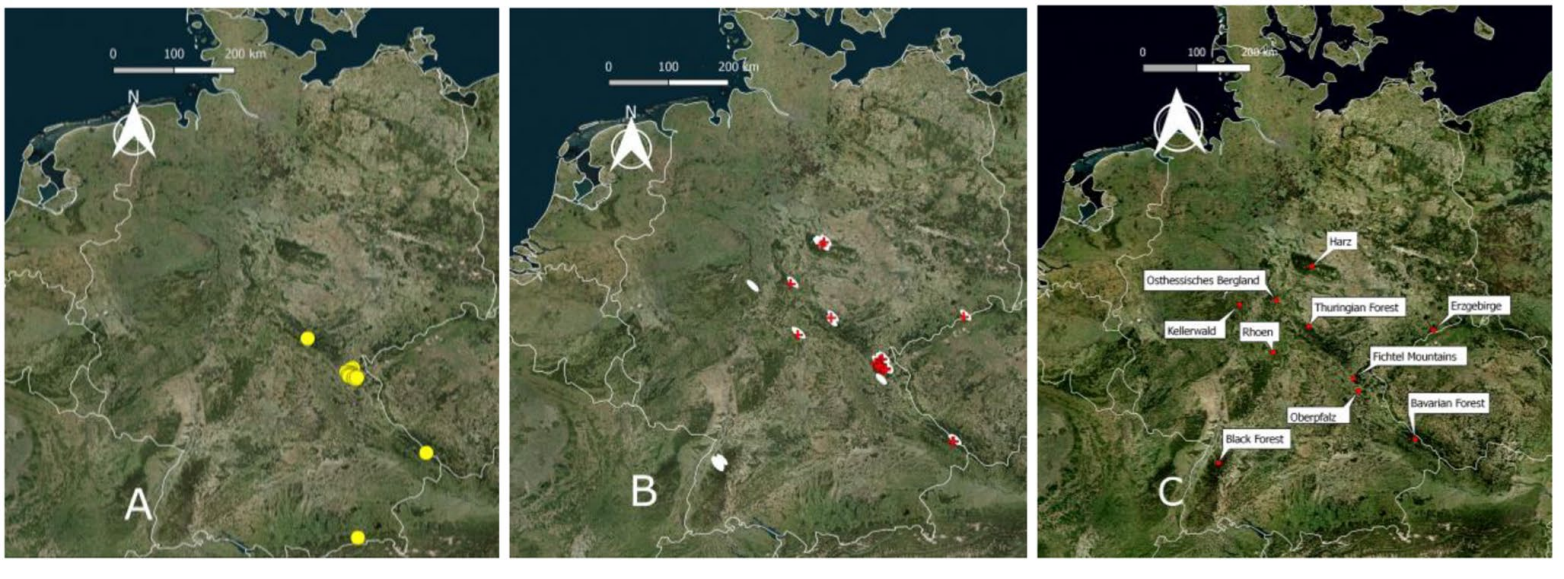
**a-c**: All known records from Germany before this study are shown by yellow dots (**a**). Our sampling sites are shown by white ellipses, new and confirmed records of *Chionea araneoides* from our study are marked by red crosses. (**b**); names of mountain ranges are provided in (**c**)

This survey is part of a larger study addressing the specialized fauna of block stone runs. In the main study more than 250 pitfall traps were installed in stone runs across 16 locations in seven low mountain ranges across Germany (Fig 1). Traps were randomly placed at each site aiming at an equal distribution across the habitat. Clearly structured stone runs consisting of source rock, slope body and base of the slope, vegetated and unvegetated areas, including basal areas overgrown by forest received the same number of traps in each area. Sampling sites received between 14 and 21 traps, diagonally distributed across the whole width.

**Fig. 2:**
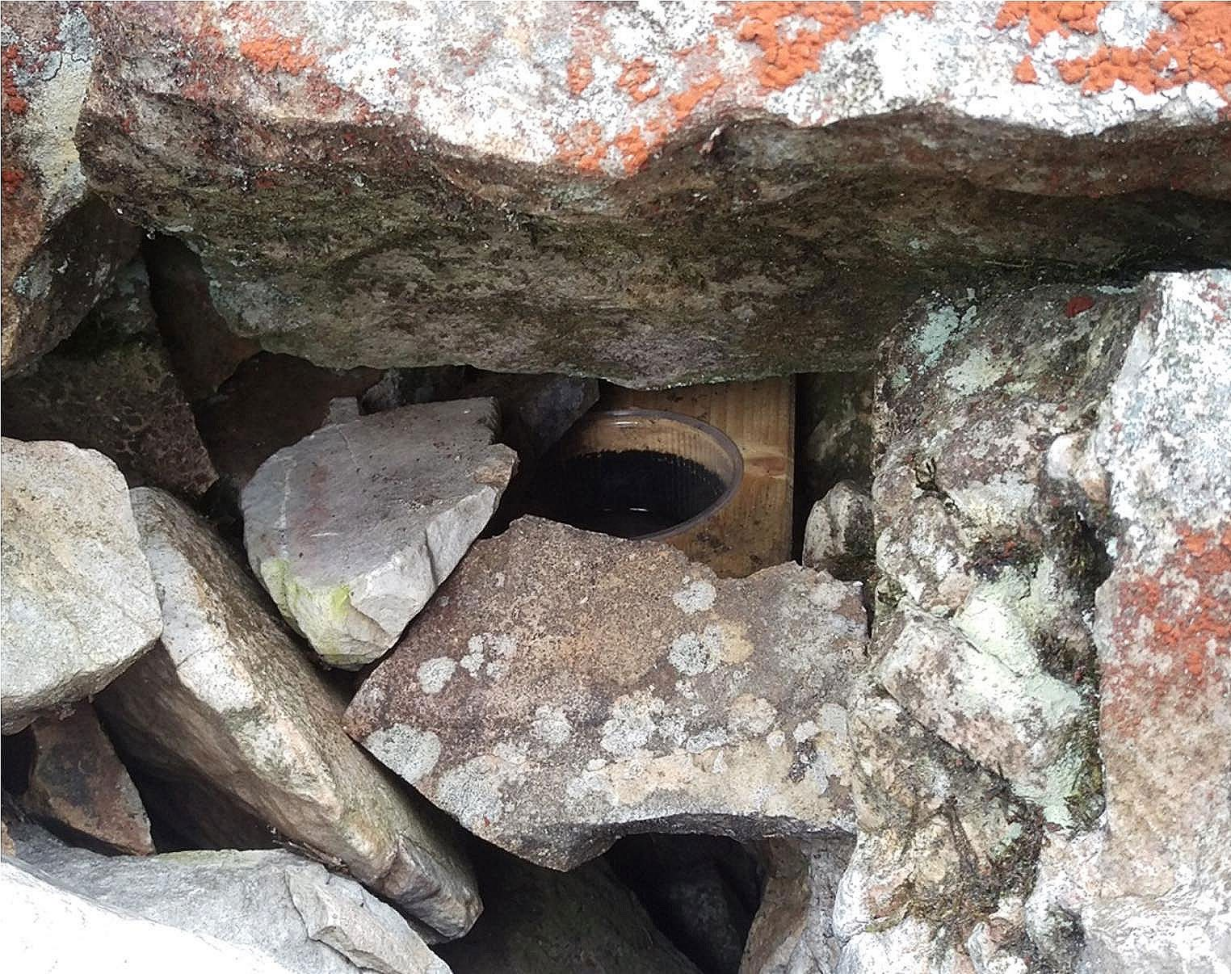
Pitfall traps were placed in a holed wooden board and put deep in the gaps between rocks

The pitfall traps consisted of a plastic cup with a volume of 0.3 or 0.5 l, placed within the rock surface aiming to provide a more or less plain contact zone to the pitfall opening, or by inserting them in a cavity or in loose substrate between the stones or by placing wooden boards with a hole for the cup between the stones (Fig. 2). Traps were filled with 99% propylene glycol (BayWa AG, Munich, Germany) and some drops of detergent. Propylene glycol has been suggested as good DNA conservation fluid for pitfall traps in the past (Höfer et al. 2015; Weigand et al. 2021), also providing the advantage of lower evaporation compared to ethanol and water based conservation fluids. Sampling was performed between April 2018 and July 2020. All traps were employed for at least one year in the stone runs. Material was collected and conservation liquid was changed every two to three months. The traps were active for the whole winter season under snow. Traps were emptied, as soon as the snow melt allowed save access to the traps.

In addition to the main sampling from the larger study, we here considered snow flies from seven additional stone runs in the Black Forest (Baden-Württemberg, South-Western Germany) and older material from several locations in the Fichtel Mountains and the Bavarian Forest (Bavaria, South-Eastern Germany), Kellerwald and Hoher Meißner (Hesse, Mid-Western Germany). The sampling in the Fichtel Mountains and Lusen Mountain in Bavaria took place from October 2008 to October 2009 as part of a project on the arthropod fauna of stony debris/talus habitats, which focused on spiders and carabids (Fritze and Blick 2010). The sampling in the northern Black Forest took place from June to September 2017 and focused on faunistic questions on spiders (Höfer et al. 2019). In the winter period the traps in all sampling sites in Black Forest were reduced to two barber traps and two soil photo eclectors. In Kellerwald sampling took place from 2014 to 2015, at Hoher Meißner from 2017 to 2018.

Additionally, some material was obtained from natural history collections to supplement the genetic dataset and to add additional records for Germany. A list of all specimens used in this study is provided in Table 1.

**Table 1:** Overview over all sequences of this study

BOLD ID/Genbank ID*	Sample ID	COI-5P Seq. Length	Genus	Species	Institution storing/Source
CHION001-23	ChioBH1	487[1n]	*Chionea*	*araneoides*	Private Collection of Robert Klesser
CHION002-23	ChioBH2	487[0n]	*Chionea*	*araneoides*	Private Collection of Robert Klesser
CHION003-23	ChioBH3	487[0n]	*Chionea*	*araneoides*	Private Collection of Robert Klesser
CHION004-23	ChioBH4	487[0n]	*Chionea*	*araneoides*	Private Collection of Robert Klesser
CHION005-23	ChioBH5	487[3n]	*Chionea*	*araneoides*	Private Collection of Robert Klesser
CHION006-23	ChioBH6	487[0n]	*Chionea*	*araneoides*	Private Collection of Robert Klesser
CHION007-23	ChioBH8	487[2n]	*Chionea*	*araneoides*	Private Collection of Robert Klesser
CHION008-23	CLUS4	487[0n]	*Chionea*	*araneoides*	Private Collection of Robert Klesser
CHION009-23	CLUS5	487[0n]	*Chionea*	*araneoides*	Private Collection of Robert Klesser
CHION010-23	CLUS6	487[0n]	*Chionea*	*araneoides*	Private Collection of Robert Klesser
CHION011-23	CLUS7	487[0n]	*Chionea*	*araneoides*	Private Collection of Robert Klesser
CHION012-23	CLUS8	487[0n]	*Chionea*	*araneoides*	Private Collection of Robert Klesser
CHION013-23	CLUS9	487[0n]	*Chionea*	*araneoides*	Private Collection of Robert Klesser
CHION014-23	CLUS11	487[0n]	*Chionea*	*araneoides*	Private Collection of Robert Klesser
CHION015-23^1^	CARA4	487[0n]	*Chionea*	*belgica*	Regional Museum of Lapland
CHION016-23	Chio1	487[0n]	*Chionea*	*lutescens*	Private Collection of Robert Klesser
CHION017-23	Chio4	487[0n]	*Chionea*	*araneoides*	Private Collection of Robert Klesser
CHION018-23	Chio13	487[0n]	*Chionea*	*araneoides*	Private Collection of Robert Klesser
CHION019-23	Chio15	487[0n]	*Chionea*	*araneoides*	Private Collection of Robert Klesser
CHION020-23	Chio37	487[0n]	*Chionea*	*araneoides*	Private Collection of Robert Klesser
CHION021-23	Chio39	487[0n]	*Chionea*	*araneoides*	Private Collection of Robert Klesser
CHION022-23	Chio51	487[0n]	*Chionea*	*lutescens*	Private Collection of Robert Klesser
CHION023-23	Chio64	487[0n]	*Chionea*	*araneoides*	Private Collection of Robert Klesser
CHION024-23	Chio66	487[0n]	*Chionea*	*araneoides*	Private Collection of Robert Klesser
CHION025-23	Chio67	487[0n]	*Chionea*	*araneoides*	Private Collection of Robert Klesser
CHION026-23	Chio68	487[0n]	*Chionea*	*araneoides*	Private Collection of Robert Klesser
CHION027-23	Chio69	487[0n]	*Chionea*	*araneoides*	Private Collection of Robert Klesser
CHION028-23	Chio70	487[0n]	*Chionea*	*araneoides*	Private Collection of Robert Klesser
CHION029-23	Chio71	487[0n]	*Chionea*	*araneoides*	Private Collection of Robert Klesser
CHION030-23	Chio72	487[0n]	*Chionea*	*araneoides*	Private Collection of Robert Klesser
CHION031-23	Chio73	487[0n]	*Chionea*	*araneoides*	Private Collection of Robert Klesser
CHION032-23	Chio74	487[0n]	*Chionea*	*araneoides*	Private Collection of Robert Klesser
CHION033-23	Chio75	487[0n]	*Chionea*	*araneoides*	Private Collection of Robert Klesser
CHION034-23	Chio81	487[0n]	*Chionea*	*araneoides*	Private Collection of Robert Klesser
CHION035-23	Chio82	487[0n]	*Chionea*	*araneoides*	Private Collection of Robert Klesser
CHION036-23	Chio88	487[0n]	*Chionea*	*araneoides*	Private Collection of Robert Klesser
CHION037-23	Chio94	487[0n]	*Chionea*	*lutescens*	Private Collection of Robert Klesser
CHION038-23	Chio101	487[0n]	*Chionea*	*araneoides*	Private Collection of Robert Klesser
CHION039-23	Chio107	487[0n]	*Chionea*	*araneoides*	Private Collection of Robert Klesser
CHION040-23	Chio115	487[0n]	*Chionea*	*araneoides*	Private Collection of Robert Klesser
CHION041-23	Chio127	487[0n]	*Chionea*	*araneoides*	Private Collection of Robert Klesser
CHION042-23	Chio137	487[0n]	*Chionea*	*belgica*	Private Collection of Robert Klesser
CHION043-23	Chio139	487[0n]	*Chionea*	*araneoides*	Private Collection of Robert Klesser
CHION044-23	Chio140	487[0n]	*Chionea*	*lutescens*	Private Collection of Robert Klesser
CHION045-23	Chio141	486[0n]	*Chionea*	*belgica*	Private Collection of Robert Klesser
CHION046-23	Chio142	487[0n]	*Chionea*	*belgica*	Private Collection of Robert Klesser
CHION047-23	Chio160	487[0n]	*Chionea*	*lutescens*	Private Collection of Robert Klesser
CHION048-23	Chio161	487[0n]	*Chionea*	*araneoides*	Private Collection of Robert Klesser
CHION049-23	Chio162_1	487[0n]	*Chionea*	*araneoides*	Private Collection of Robert Klesser
CHION050-23	Chio162_2	487[0n]	*Chionea*	*araneoides*	Private Collection of Robert Klesser
CHION051-23	Chio164	487[0n]	*Chionea*	*araneoides*	Private Collection of Robert Klesser
CHION052-23	Chio165	487[0n]	*Chionea*	*araneoides*	Private Collection of Robert Klesser
CHION053-23	Chio166	487[0n]	*Chionea*	*araneoides*	Private Collection of Robert Klesser
CHION054-23	Chio167	486[0n]	*Chionea*	*belgica*	Private Collection of Robert Klesser
CHION055-23	Chio168	487[0n]	*Chionea*	*araneoides*	Private Collection of Robert Klesser
CHION056-23	Chio172	487[0n]	*Chionea*	*araneoides*	Private Collection of Robert Klesser
CHION057-23	Chio174	487[0n]	*Chionea*	*araneoides*	Private Collection of Robert Klesser
CHION058-23	Chio175	487[0n]	*Chionea*	*araneoides*	Private Collection of Robert Klesser
CHION059-23	Chio176	487[0n]	*Chionea*	*belgica*	Private Collection of Robert Klesser
CHION060-23	Chio177	486[0n]	*Chionea*	*belgica*	Private Collection of Robert Klesser
CHION061-23	Chio178	486[0n]	*Chionea*	*belgica*	Private Collection of Robert Klesser
CHION062-23	Chio183	487[0n]	*Chionea*	*belgica*	Private Collection of Robert Klesser
CHION063-23	Chio189	487[0n]	*Chionea*	*araneoides*	Private Collection of Robert Klesser
CHION064-23	Chio190	487[0n]	*Chionea*	*araneoides*	Private Collection of Robert Klesser
CHION065-23	Chio191	487[0n]	*Chionea*	*araneoides*	Private Collection of Robert Klesser
CHION066-23	Chio192	487[0n]	*Chionea*	*araneoides*	Private Collection of Robert Klesser
CHION067-23	Chio194	487[0n]	*Chionea*	*lutescens*	Private Collection of Robert Klesser
CHION068-23	Chio195	487[0n]	*Chionea*	*araneoides*	Private Collection of Robert Klesser
CHION069-23	Chio199	487[0n]	*Chionea*	*araneoides*	Private Collection of Robert Klesser
CHION070-23	Chio200	487[0n]	*Chionea*	*araneoides*	Private Collection of Robert Klesser
CHION071-23	Chio201	487[0n]	*Chionea*	*araneoides*	Private Collection of Robert Klesser
CHION072-23	Chio202	487[0n]	*Chionea*	*araneoides*	Private Collection of Robert Klesser
CHION073-23	Chio203	487[0n]	*Chionea*	*araneoides*	Private Collection of Robert Klesser
CHION074-23	Chio204	487[0n]	*Chionea*	*araneoides*	Private Collection of Robert Klesser
CHION075-23	Chio205	487[0n]	*Chionea*	*araneoides*	Private Collection of Robert Klesser
CHION076-23	Chio206	487[0n]	*Chionea*	*araneoides*	Private Collection of Robert Klesser
CHION077-23	Chio207	487[0n]	*Chionea*	*araneoides*	Private Collection of Robert Klesser
CHION078-23	Chio208	487[0n]	*Chionea*	*araneoides*	Private Collection of Robert Klesser
CHION079-23	Chio210	487[0n]	*Chionea*	*araneoides*	Private Collection of Robert Klesser
CHION080-23	Chio211	487[0n]	*Chionea*	*araneoides*	Private Collection of Robert Klesser
CHION081-23	Chio212	487[0n]	*Chionea*	*araneoides*	Private Collection of Robert Klesser
CHION082-23	Chio213	487[0n]	*Chionea*	*araneoides*	Private Collection of Robert Klesser
CHION083-23	Chio214	487[0n]	*Chionea*	*araneoides*	Private Collection of Robert Klesser
CHION084-23	Chio216	487[0n]	*Chionea*	*araneoides*	Private Collection of Robert Klesser
CHION085-23	Chio218	487[0n]	*Chionea*	*araneoides*	Private Collection of Robert Klesser
CHION086-23	Chio219	487[0n]	*Chionea*	*araneoides*	Private Collection of Robert Klesser
CHION087-23	Chio220	487[0n]	*Chionea*	*araneoides*	Private Collection of Robert Klesser
CHION088-23	Chio221	487[0n]	*Chionea*	*araneoides*	Private Collection of Robert Klesser
CHION089-23	Chio0046	487[0n]	*Chionea*	*araneoides*	Regional Museum of Lapland
CHION090-23	Chio_Bad_Baiersdorf	487[0n]	*Chionea*	*lutescens*	Private Collection of Robert Klesser
CHION091-23^1^	CLUT_TUZ075533	487[0n]	*Chionea*	*lutescens*	University of Tartu, Natural History Museum
CHION092-23^1^	CLUT_TUZ254688	487[0n]	*Chionea*	*lutescens*	University of Tartu, Natural History Museum
HQ982416^2^			*Cladura*	*flavoferruginea*	Sequence from Genbank
KR970516^2^			*Cladura*	*flavoferruginea*	Sequence from Genbank

^1^Extraction from loan

^2^Sequence from Database

To provide a summary of the current knowledge on the distribution of *C. araneoides* in Germany, all records were added to a map layer and visualized in QGIS 2.18 (QGIS Development Team 2016) on a Bing Satellite map (via QuickMapsServices Plugin, 24th Oct. 2023). Administration layers of German boarders and boarders of federal states were added from GADM database (www.gadm.org). Cases of sympatric distributions of *C. araneoides* and *C. belgica* and *C. lutescens* were mapped in two areas in more detail.

### Identification and DNA barcoding

Morphological determinations were performed using the key and description in Oosterbroek and Reusch (2008). Antenna with 9–10 segments and a sclerotized lobe at base of the gonostyle in males were the main traits we focused on to distinguish *C. araneoides* from all other congenerics. To delimit *C. lutescens* and *C. belgica*, the sternite 9 and a (missing) medial comb, most often with a row of fine bristles along the hind margin were considered.

### DNA barcoding

In addition, we performed DNA barcoding of a subset of specimens (Table 1). We used a standard CTAB protocol (Borges et al. 2009) for non-invasive DNA extraction: after the lysis we retrieved the specimens from the buffer and stored them in ethanol. The barcoding fragment of the COI gene was amplified using standard PCR procedures with the primers LCO-1490 and HCO-2198 (Folmer et al. 1994). PCR was performed with the following setup: 5.7 μl PCR grade water, 2 μl 5x buffer, 0.5 μl of each primer (10 μM), 0.2 μl dNTPs (10 mM), 0.1 μl DreamTaq™ polymerase and 1 μl template. PCR conditions were as follows: activation at 95 °C for 5 min, followed by 35 cycles of 30 s denaturation at 95 °C, 1 min annealing at 50 °C and 1 min elongation at 72 °C. Finally, a 10 min elongation step was performed. PCR success was checked on 1.5% agarose gels stained with GelGreen. Successful products were purified with an enzyme mix consisting of Exonuclease I and Shrimp-Alkaline Phosphatase (ExoSap). Products were sent to Macrogen (Amsterdam, Netherlands) for sequencing.

### Analyses

All chromatograms were checked, trimmed and proofread in GENEIOUS v.9 (Kearse et al. 2012). MUSCLE (Edgar 2004), as implemented in GENEIOUS, was used to align all sequences. The resulting alignments were trimmed to similar length. Overall, we submitted 92 new sequences to BOLD (Ratnasingham and Hebert 2007): 74 of *C. araneoides*, 9 of *C. belgica* and 9 of *C. lutescens* (Table 1). We added two sequences of *Cladura flavoferruginea* (KR970516, HQ982416) from GenBank (Geer et al. 2009) as outgroups. The final alignment reached a length of 480 bp.

We performed tree reconstruction with BEAST v. 2.6.2 (Bouckaert et al. 2014). The best substitution model, JC69, was determined using the R package PHANGORN (Schliep et al. 2017) in mran v.3.4 (Microsoft 2017) with RStudio v.1.0.143 (RStudio Team 2015). BEAST input files were generated in BEAUti 2.6.2 (Bouckaert et al. 2014), running a chain length of 10 Mio. The MCMC tree file was checked with Tracer 1.7 (Rambaut et al. 2018) for convergence and ESS (Effective Sample Size) values > 200. We performed three runs, all provided consistent results. The most plausible tree was chosen by TreeAnnotator v.2.6.2 (Bouckaert et al. 2014) after excluding a burn-in of 10%. The resulting single tree was visualized by iTOL (https://itol.embl.de/, 2021).

In a further step, a locations- and sequence file was generated and linked to the tree on a geographic map in GenGis v. 2 (Parks et al. 2013). The tree was reduced to a subtree, which only contained *C. araneoides*. Finally, the basic alignment was reduced to *C. araneoides* sequences to construct a haplotype network in PopArt v. 1.4 (Leigh and Bryant 2015).

Further, we calculated the p-distance between the main clades of *C. araneoides* in in MEGA 11 (Tamura et al. 2021), both lineages detected in phylogenetic analyses were defined as subsets. Finally, we used different species delimitation models to determine potential mOTUs (molecular operational taxonomic units). For the online versions of ASAP (Puillandre et al. 2021) and ABGD (Puillandre et al. 2012), we used the alignment file as input after removing outgroups. ABGD was run with standard parameters (10 Steps, X = 1.5, JC69) after testing different parameters without any effects on the results. In ASAP we tested all substitution models, also without any effects on clusters. Hence, we used the JC69. For PTP and bPTP (Zhang et al. 2013) we also used the online versions with standard parameters (100.000 MCMC generations, thinning = 100, burn-in 10%). Treefiles were used as input, outgroups were cropped. Single threshold GMYC (*st*GMYC) and multiple threshold GMYC (Pons et al. 2006) (*mt*GMYC) analyses were performed with Microsoft R mRAN v.3.4 (Microsoft 2017) in RStudio 2021.09.1 + 372 “Ghost Orchid” (RStudio Team 2015) and the packages Ape (Paradis et al. 2004), Splits (Ezard et al. 2009), Paran (Dinno 2012) and Mass (Venables and Ripley 2002).

## Results

### DNA barcoding, species identity and distribution data

Overall, we recorded 248 *C. araneoides* from 10 stone runs in Germany (for details see Table 2) from our traps and added more than 2000 specimens from former studies to our dataset. Our morphological identification resulted only in *C. araneoides* and *C. lutescens* following the key of Oosterbroek and Reusch (2008). However, as we did not perform gentialia preparations, we were not able to distinguish *C. belgica* from *C. lutescens*. Hence, all records of these two, for which morphological identification was not confirmed by barcoding, were excluded. However, since our focus was *C. araneoides* this is not relevant for our main analysis. The comparisons of the barcodes to the reference databases BOLD (Ratnasingham and Hebert 2007) and GenBank (Geer et al. 2009) supported all identifications of *C. araneoides*.

**Table 2:** Sampling data of all locations of stone runs und rock habitats

Region	Site	Federal State	Lon	Lat	♂	♀	Alti-tude (m)	Year (start)	Year (end)	Method	Duration	Species
Bavarian Forest	Lusen Moor	Bavaria	13.50845	48.94195	31	24	1297	2009	2009	Pitfall Traps	1 Year	*Chionea araneoides*
Bavarian Forest	Lusen	Bavaria	13.5068	48.9399	151	146	1313	2008	2009	Pitfall Trapss	1 Year	*Chionea araneoides*
Bavarian Forest	Lusen	Bavaria	13.50626	48.939142	61	32	1313	2019	2020	Pitfall Traps	1 Year	*Chionea araneoides*
Bavarian Forest	Waldweg Waldhaeuser-Lusen	Bavaria	10.5691	10.5691	2	1	950	2020	2020	Hand Capture	5 Days	*Chionea araneoides*
Bavarian Forest	Teufelsloch	Bavaria	13.481575	48.9395	1		1084	2019	2020	Hand Capture	1 Year	*Chionea araneoides*
Erzgebirge	Kahleberg	Saxony	13.72997	50.749047	13	8	898	2018	2019	Pitfall Traps	1 Year	*Chionea araneoides*
Fichtel Mountain	Ochsenkopf Nord	Bavaria	11.80925	50.0317	7	4	1000	2018	2019	Pitfall Traps	1 Year	*Chionea araneoides*
Fichtel Mountain	Backöfele	Bavaria	11.8552	50.05585	10	5	1020	2018	2019	Pitfall Traps	1 Year	*Chionea araneoides*
Fichtel Mountains	Epprechstein	Bavaria	11.9157	50.1448	15	4	780	2008	2009	Pitfall Traps	1 Year	*Chionea araneoides*
Fichtel Mountains	Braurangenbruch	Bavaria	11.90185	50.1479	12	8	760	2008	2009	Pitfall Traps	1 Year	*Chionea araneoides*
Fichtel Mountains	Gr. Waldstein, Fels	Bavaria	11.8533	50.1287	7	0	845	2008	2009	Pitfall Traps	1 Year	*Chionea araneoides*
Fichtel Mountains	Gottmannsberg/Gefrees	Bavaria	11.7623	50.0894	16	10	640	2008	2009	Pitfall Traps	1 Year	*Chionea araneoides*
Fichtel Mountains	Rudolfstein	Bavaria	11.8777	50.0722	46	21	820	2008	2009	Pitfall Traps	1 Year	*Chionea araneoides*
Fichtel Mountains	Quacke1, Fels	Bavaria	11.8727	50.0701	25	9	820	2008	2009	Pitfall Traps	1 Year	*Chionea araneoides*
Fichtel Mountains	Drei Brüder, Fels	Bavaria	11.8719	50.0694	32	13	825	2008	2009	Pitfall Traps	1 Year	*Chionea araneoides*
Fichtel Mountains	Hohe Heide, Wald	Bavaria	11.8052	50.0705	3	0	845	2008	2009	Pitfall Traps	1 Year	*Chionea araneoides*
Fichtel Mountains	Schneebg-Haberstein	Bavaria	11.84375	50.04475	128	81	840	2008	2009	Pitfall Traps	1 Year	*Chionea araneoides*
Fichtel Mountains	Schneeberg 2	Bavaria	11.8483	50.0657	54	36	980	2008	2009	Pitfall Traps	1 Year	*Chionea araneoides*
Fichtel Mountains	Schneeberg 5 (Backoef.)	Bavaria	11.8552	50.05585	121	94	1020	2008	2009	Pitfall Traps	1 Year	*Chionea araneoides*
Fichtel Mountains	Nußhardt, Felsen	Bavaria	11.86625	50.03995	51	32	950	2008	2009	Pitfall Traps	1 Year	*Chionea araneoides*
Fichtel Mountains	Seehügel	Bavaria	11.8717	50.0283	105	36	905	2008	2009	Pitfall Traps	1 Year	*Chionea araneoides*
Fichtel Mountains	Platte	Bavaria	11.8902	50.0167	70	50	860	2008	2009	Pitfall Traps	1 Year	*Chionea araneoides*
Fichtel Mountains	Ochsenkopf, Goethefelsen	Bavaria	11.8121	50.0286	34	15	920	2008	2009	Pitfall Traps	1 Year	*Chionea araneoides*
Fichtel Mountains	Ochsenkopf North	Bavaria	11.80925	50.0317	97	61	1000	2008	2009	Pitfall Traps	1 Year	*Chionea araneoides*
Fichtel Mountains	Ochsenkopf, South	Bavaria	11.8258	50.0286	79	42	980	2008	2009	Pitfall Traps	1 Year	*Chionea araneoides*
Fichtel Mountains	Vord. Ringberg	Bavaria	11.921	49.997	21	4	760	2008	2009	Pitfall Traps	1 Year	*Chionea araneoides*
Fichtel Mountains	Hohe Matze	Bavaria	11.932	49.998	96	25	795	2008	2009	Pitfall Traps	1 Year	*Chionea araneoides*
Fichtel Mountains	Koesseine	Bavaria	11.9784	49.9881	72	28	895	2008	2009	Pitfall Traps	1 Year	*Chionea araneoides*
Fichtel Mountains	Luisenburg, Rock	Bavaria	11.9964	50.0102	40	26	810	2008	2009	Pitfall Traps	1 Year	*Chionea araneoides*
Fichtel Mountains	Koesseine (Forest)	Bavaria	11.981343	49.987212	4	1	915	2018	2019	Pitfall Traps	1 Year	*Chionea araneoides*
Harz	Hammersteinklippe	Lower Saxony	10.45039	51.76656		3	760	1999	2000	Pitfall Trap	7 Months	*Chionea araneoides*
Harz	Achtermann	Lower Saxony	10.5691	51.762	4	5	925	2019	2020	Hand Capture		*Chionea araneoides*
Harz	Brocken	Saxony-Anhalt	10.615771	51.801587	51	37	1120	2018	2019	Hand Capture	2 Days	*Chionea araneoides*
Harz	Brocken	Saxony-Anhalt	10.615771	51.801587	1	2	1120	2020	2021	Hand Capture	2 Days	*Chionea araneoides*
Osthessisches Bergland	Hoher Meißner	Hessen	9.87438	51.21676	101	52	665	2017	2018	Pitfall Traps	1 Year	*Chionea araneoides*
Rhoen	Stirnberg 14	Bavaria	10.03115	50.49195	10	7	881	2004	2005	Pitfall Traps	1 Year	*Chionea araneoides*
Thuringian Forest	Luetsche, Block forest	Thuringia	10.773647	50.73639		1	560	2018	2019	Pitfall Traps	1 Year	*Chionea araneoides*
Black Forest	Seebach	Baden-Wuerttemberg	8.18	48.59				2017	2018	2 Pitfall Traps + Photo Ground Eclectors	16th Oct 2017–17th April.2018	*Chionea lutescens and/or belgica*
Black Forest	Altsteigerskopf	Baden-Wuerttemberg	8.22	48.58				2017	2018	2 Pitfall Traps + Photo Ground Eclectors	16th Oct 2017–17th April.2018	*Chionea lutescens and/or belgica*
Black Forest	Ochsenkopf	Baden-Wuerttemberg	8.3	48.64				2017	2018	2 Pitfall Traps + Photo Ground Eclectors	16th Oct 2017–17th April.2018	*Chionea lutescens and/or belgica*
Black Forest	Melkereikopf	Baden-Wuerttemberg	8.2	48.55				2017	2018	2 Pitfall Traps + Photo Ground Eclectors	16th Oct 2017–17th April.2018	*Chionea lutescens and/or belgica*
Black Forest	Seibelseckle	Baden-Wuerttemberg	8.22	48.59				2017	2018	2 Pitfall Traps + Photo Ground Eclectors	16th Oct 2017–17th April.2018	*Chionea lutescens and/or belgica*
Black Forest	Hornisgrinde	Baden-Wuerttemberg	8.2	48.61				2017	2018	2 Pitfall Traps + Photo Ground Eclectors	16th Oct 2017–17th April.2018	*Chionea lutescens and/or belgica*
Harz	Odertal	Lower Saxony	10.55916	51.73684			590	2018	2019	Pitfall Traps	1 Year	*Chionea lutescens and/or belgica*
Harz	Mönchskappenklippe	Lower Saxony	10.46256	51.74676			667	2018	2019	Pitfall Traps	1 Year	*Chionea lutescens and/or belgica*
Harz	Hammersteinklippe	Lower Saxony	10.45039	51.76656			760	2018	2019	Pitfall Traps	1 Year	*Chionea lutescens and/or belgica*
Harz	Wolfswarte	Lower Saxony	10.50266	51.79065			910	2018	2019	Pitfall Traps	1 Year	*Chionea lutescens and/or belgica*
Kellerwald	Banfe	Hesse	8.97	51.17			280	2014	2015	Pitfall Traps		*Chionea lutescens and/or belgica*
Kellerwald	Daudenberg	Hesse	8.99	51.16			425	2014	2015	Pitfall Traps		*Chionea lutescens and/or belgica*
Oberpfalz	Rauher Kulm	Bavaria	11.849721	49.828477			665	2019	2020	Pitfall Traps	1 Year	*Chionea lutescens and/or belgica*
Rhoen	Schafstein	Hesse	9.972061	50.503631			805	2018	2019	Pitfall Traps	1 Year	*Chionea lutescens and/or belgica*
Thuringian Forest	Großer Beerberg	Thuringia	10.741893	50.665314			889	2018	2019	Pitfall Traps	1 Year	*Chionea lutescens and/or belgica*

Determinations of loaned specimens from the Naturkundemuseum Stuttgart (SMNS) and the Zoologische Staatssammlung München (ZSM) did not show any unknown records of *C. araneoides* from Germany. The collections of Naturkundemuseum Karlsruhe (SMNK) and Museum der Natur Hamburg (ZMH) also did not include records of *Chionea araneoides*. In the collections of the Naturkundemuseum Leipzig (NKML), we found one Individual (Collection ID NML-i2022/1556) from Erzgebirge from 20th − 27th Jan 1934 without any further information about the precise location. Overall, we found several misidentified specimens and very old specimens named *C. araneoides* from times, when all *Chionea* species were handled as *C. araneoides* without any further knowledge about other species in Germany.

### Phylogenetic and phylogeographic assessment

**Fig. 3:**
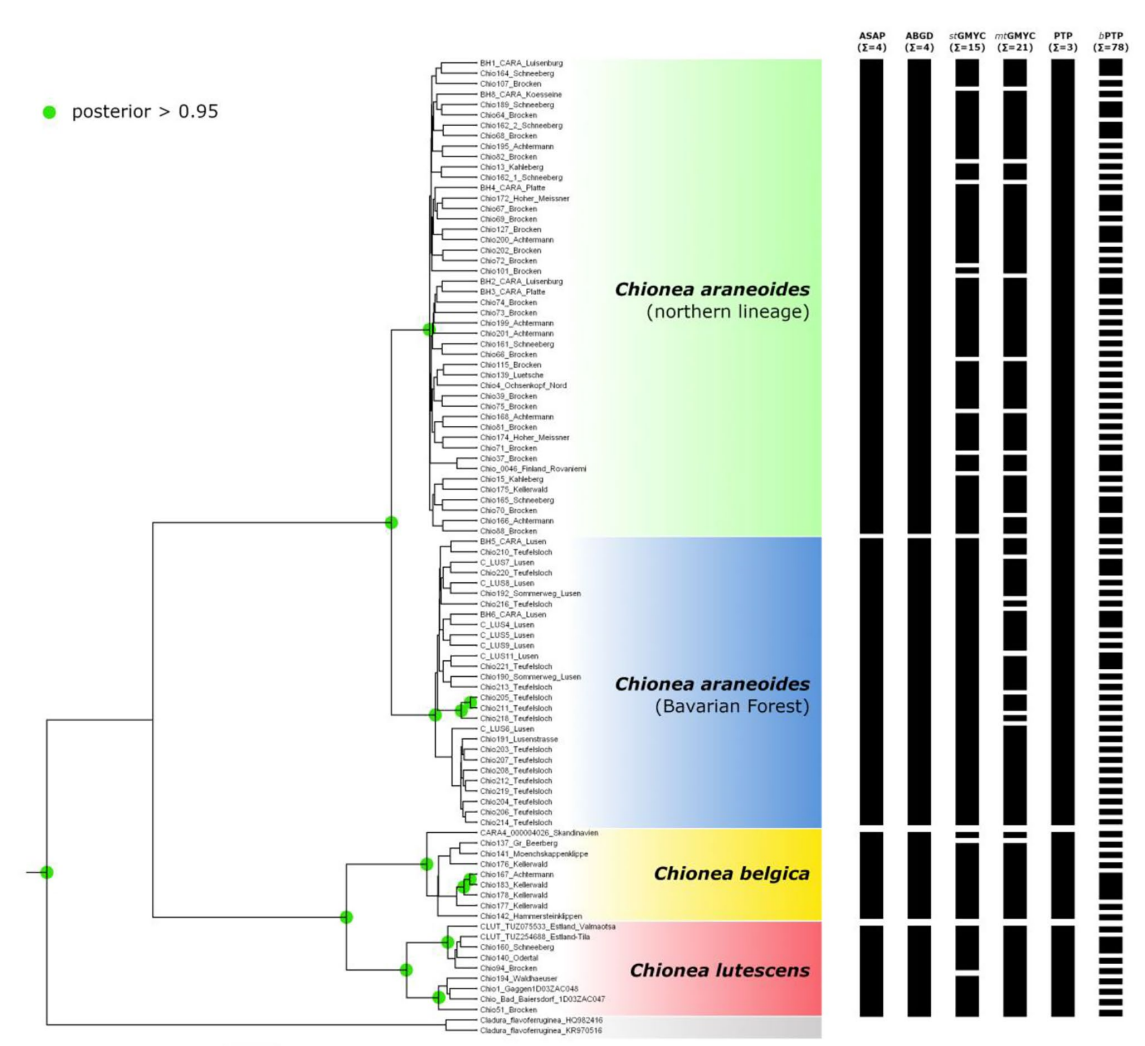
Phylogenetic tree based on COI. Two different clades of *Chionea araneoides* from the Bavarian Forest (blue) and all northern locations (green) show a deep split, almost similar to the established species *Chionea lutescens* (red) and *Chionea belgica* (yellow). The results of different species delimitation (ASAP, ABGD, stGMYC, mtGMYC, PTP, bPTP) methods are shown as black bars next to the tree. Green circles denote nodes with posterior probability (PP) greater than 0.95

**Fig. 4:**
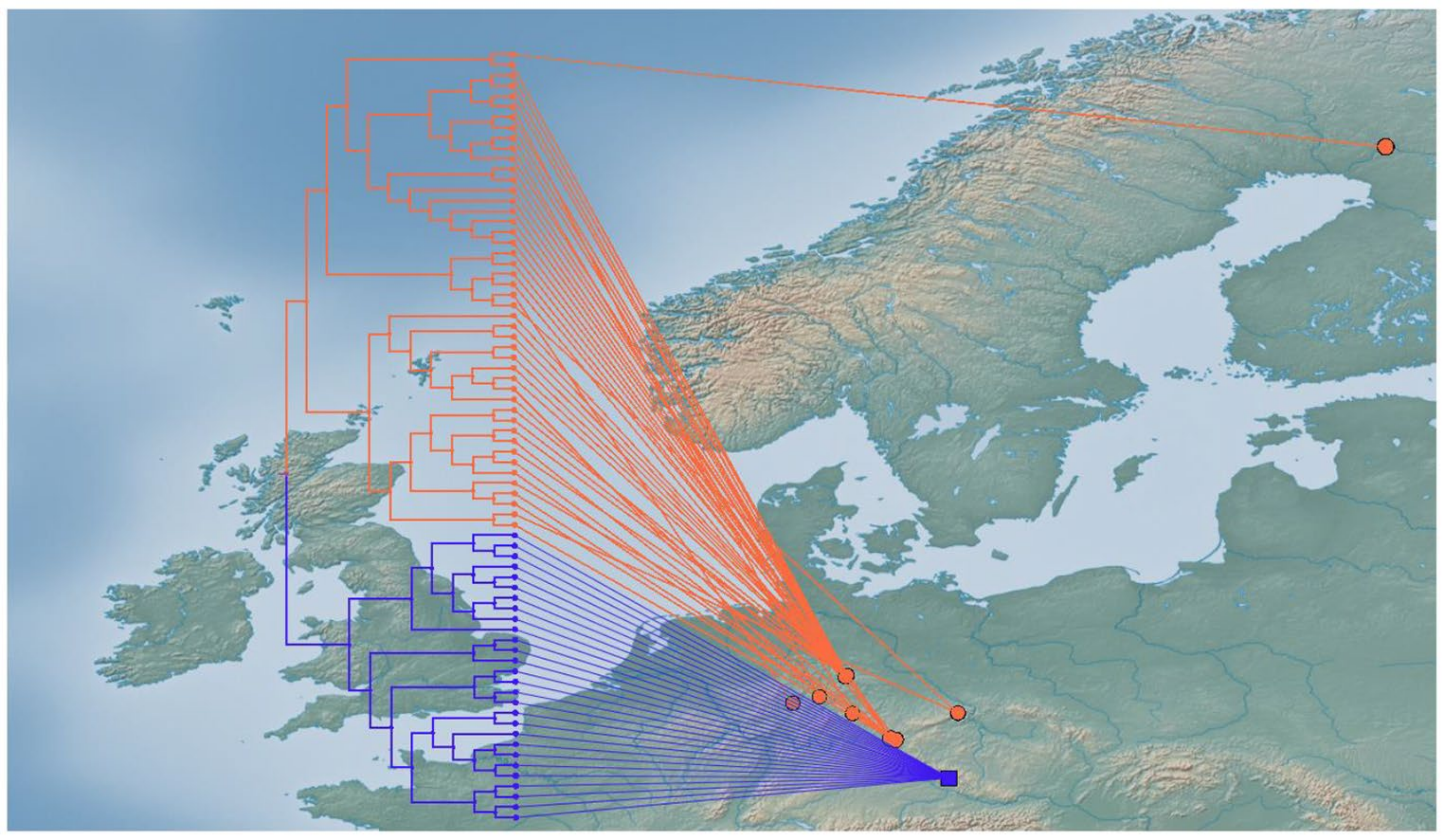
The two clades of *Chionea araneoides* are extracted from the phylogenetic tree and plotted on a map using the software GenGis. The northern lineage (pink) shows a closer relation to a sample from Finland than to the lineage from the Bavarian Forest (blue)

The phylogenetic tree (Fig. 3) based on COI showed distinct clades and well-supported splits of *C. belgica, C lutescens* (pp = 1) and *C. araneoides* (pp = 0.93). *Chionea araneoides* further split into two clearly distinct clades with high support (pp = 1). One clade only consists of specimens from the Bavarian Forest (Fig. 3), whereas the other clade comprises all other specimens from Harz, Thuringian Forest, Erzgebirge, Fichtel Mountains, Kellerwald and Finland (Fig. 4). GenGis was used to map all sequences on a European map and detected a link between the northern clade in Germany and a sample from Finland.

**Fig. 5:**
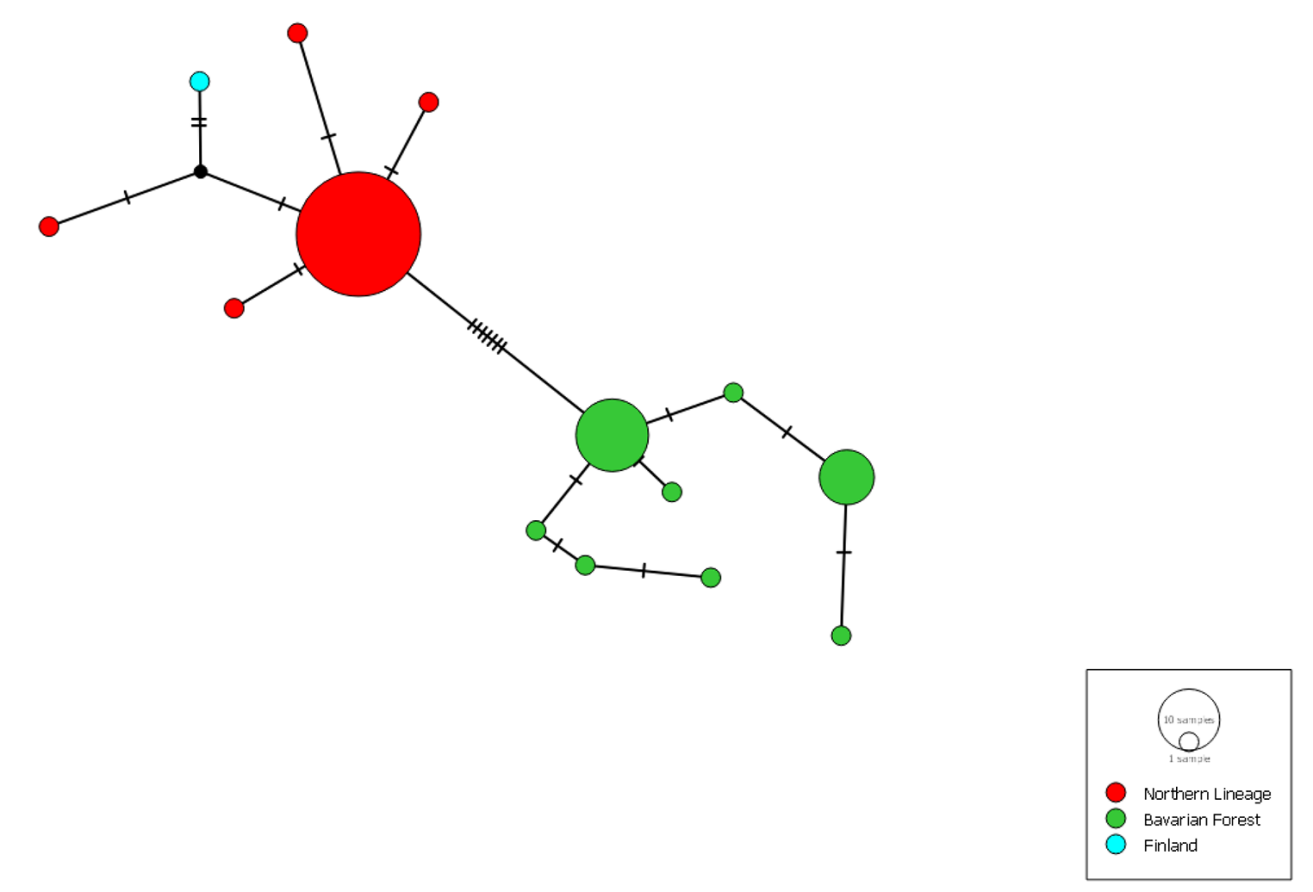
Haplotype network of northern lineage (red) with Finish sample (light blue) and lineage from Bavarian Forest (green)

The haplotype network supported the presence of two different clades, with a strong COI diversity within both clades (Fig. 5). The p-distance between the clades of *C. araneoides* from the Bavarian Forest and all other populations was 0.0466.

Species delimitation based on bPTP (78 mOTUs), stGMYC (15 mOTUs) and mtGMYC (21 mOTUs) showed oversplitting, while ASAP (4 mOTUs), ABGD (4 mOTUs), and PTP (3 mOTUs) were much more conservative. ABGD, ASAP and PTP detected *Chionea belgica* and *Chionea lutescens* accordingly to morphological determinations. ASAP and ABGD found two mOTUs within *Chionea araneoides*. A distinct Bavarian lineage was found also by stGMYC, which also suggested additional mOTUs within the northern lineage. PTP was the most conservative tool, which suggested three species in the dataset, in line with morphological determinations (Fig. 3).

### Ecological and morphological observations

Living individuals of *Chionea araneoides* were not found on days reaching temperatures above 0 °C at the snow surface. Most catches (> 100) were done by traps under snow during the winter period, protected from wind and daylight. Larvae were only found in soil under mosses between stones.

**Fig. 6:**
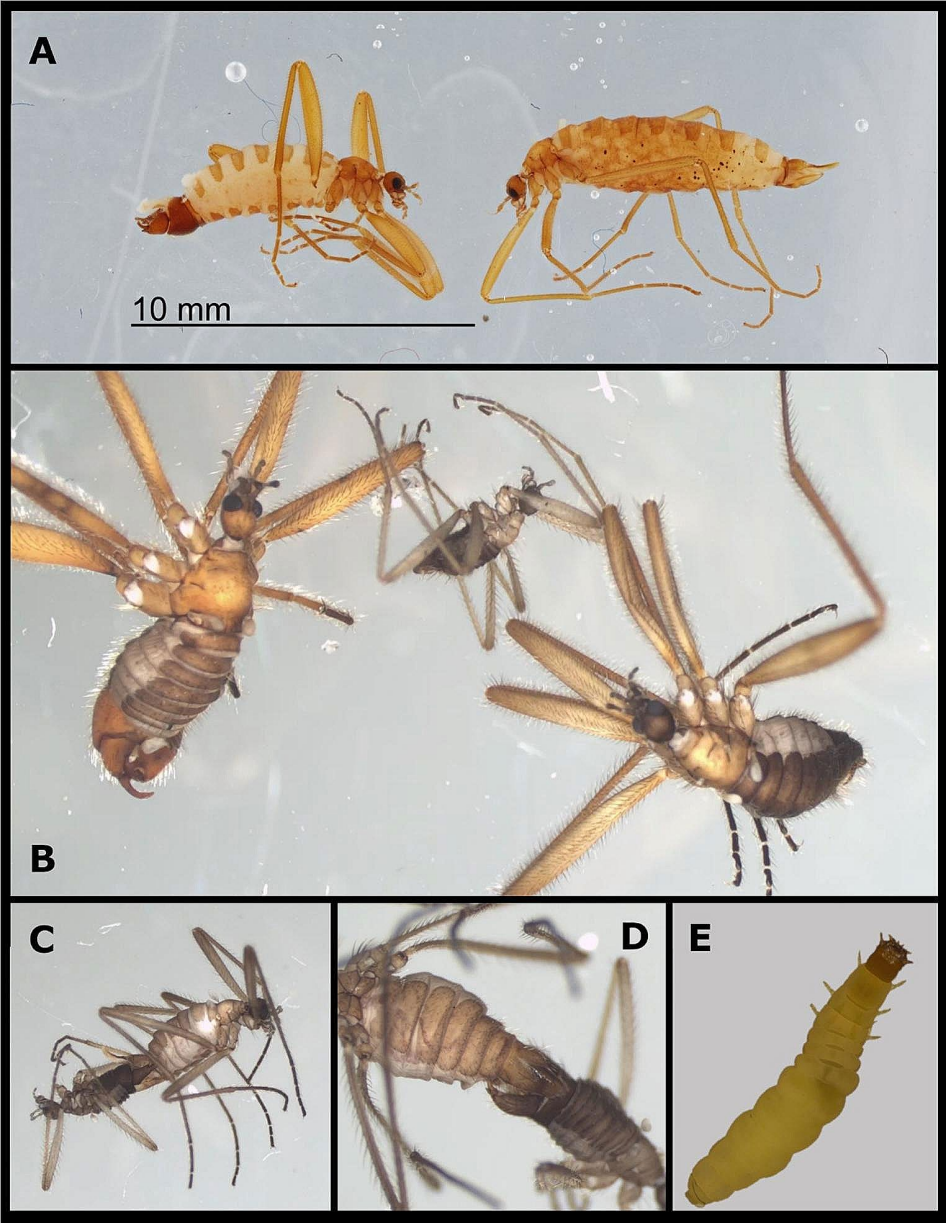
**a-e**. Different male phenotypes and sexes in *Chionea araneoides*: “normal” sized male and female of *C. araneoides* (**A**). Two types of males: *Chionea lutescens* male, *Chionea araneoides* small male, *Chionea araneoides* large male (from the left to the right) (**B**). Phenotypic small male of *Chionea araneoides* mating with normal female (**C, D**). Larval stage of *Chionea araneoides* from Haberstein (Fichtel Mountains), extracted from moss in stone run a under snow (**E**)

During identification of *Chionea* samples two types of males were found. While sizes of “normal” males are equal to female sizes (Fig. 6a) and males of other species of the genus, e.g. *C. lutescens* (Fig. 6b), the second type of males is much smaller (Fig. 6b, c, d). Nevertheless smaller males also seem to have mating success and were found in copula with normal sized females. Also larvae were found during the study in moss and soil (Fig. 6e).

## Discussion

*Chionea* snow flies represent an interesting, but largely understudied taxon of winter active flightless Diptera. Especially, *C. araneoides*, a specialist of stone runs holds the potential for interesting biogeographic patterns due to its isolated populations in Scandinavia, the Alps and the Central European lower mountain ranges connecting the main distributions. Here, we aimed to get some more insights into its distribution, biogeography and differentiation patterns using a large trapping campaign and COI barcoding data.

### New and updated records for *C. araneoides*

Our large trapping campaign yielded several new records and confirmed old findings of the stone run specialist *C. araneoides* in Germany. So far *C. araneoides* was only known from the Fichtel Mountains, the Bavarian Forest (both Blick and Fritze 2009) and the Thuringian part of the Rhoen (Bellstedt et al. 2014). We report new records for four additional low mountain ranges in Germany and four additional federal states: Erzgebirge (Saxony, 21 records from Kahleberg), Thuringian Forest (Thuringia, one specimen from Lütsche), Harz (Lower Saxony, 9 records from Achtermann & Hammersteinklippe; Saxony-Anhalt, 89 records from Brocken) and Hoher Meißner (Hesse, 154 specimens). Further, we were able to confirm records from the Fichtel Mountains (Backöfele, Ochsenkopf), the Bavarian Forest (Blick and Fritze 2009; Fritze and Blick 2010) and the Rhoen Mountains (now in the Bavarian and Hessian parts). Together with previous records the species now is documented for six federal states (Bavaria, Saxony, Saxony-Anhalt, Lower Saxony, Hesse).

### Cryptic differentiation and biogeography

We used the COI fragment, to test how well the different *Chionea* species can be differentiated and if there is further population level differentiation. Our phylogenetic analyses showed good resolution of the described species and additionally two clearly distinct lineages of *C. araneoides* in Germany. The northern lineage, which includes the populations from Fichtelgebirge up to the Harz, likely reaches up to Scandinavia: one sample from Finland clustered with this clade.

In absence of samples from the Alps and Eastern Europe, we can just raise hypotheses on the origin of the two lineages and possible colonization scenarios. One explanation are different pathways of dispersion in Germany during the last glacial. The link of the northern populations to Finland suggests a migration from Scandinavia or from a central European refuge to Scandinavia. We know such patterns from other insect species as the butterflies *Lycaena helle* (Habel et al. 2010, 2011) and the genus *Boloria* (Maresova et al. 2019). Further, we know the importance of extra-Mediterranean refugia for non-flying insects as ground beetles *Carabus irregularis* (Homburg et al. 2013) and *Carabus sylvestris* (Drees et al. 2016); isolation in such smaller refugial areas may also explain the differentiation patterns we observed. The large divergence of the two lineages suggests certainly that we have two source populations. Only additional samples from the potential ancestor populations from the Alps and more samples from Scandinavia can help to explore the history of these lineages further.

Based on the genetic distance (p-distance = 0.0466) between the lineages, they may even represent independent taxa (compare with Hebert et al. 2004). To further test this we performed different species delimitation approaches. bPTP, stGMYC and mtGMYC methods clearly determined too many mOTUs. Such patterns of oversplitting are often observed for tree based single locus species delimitations (Pentinsaari et al. 2017; Harms et al. 2018; Klesser et al. 2021). Hence, we focused on more conservative models such as ASAP, ABGD and PTP; we used the split between the known species *C. belgica* and *C. lutescens* as a calibration as these two have been widely accepted as valid species. Applying the methods to *C. araneoides* yielded unequivocal results with models alternating between one (PTP) or two mOTUs (ASAP, ABGD) for *C. araneoides*. This suggests, that the two clearly separated (pp ≥ 0.95) clades within *C. araneoides* represent diverging lineages.

In general, the level of genetic distance of COI observed between the two lineages is high, potentially justifying species status. Considering that records are only known from stone runs and rock systems in central Europe, it is likely that the species represents a glacial relict here and populations have been strictly isolated for a long time. Hence, the observed divergence may suggest the presence of isolated allopatric taxa, or at least populations on the way of becoming distinct species. To fully solve their taxonomic status, detailed morphological and further genetic analyses are required. However, registering the two lineages as separate evolutionary significant units (ESUs) may become important for conservation as cold adapted species may face severe threats by global climate change.

### Ecological observations

*Chionea araneoides* is known for preferring temperatures around − 3.5 °C (Sömme and Östbye 1969) with a range from − 6 °C up to 0 °C and barely above. They can be found on snow, usually on windless days (Hågvar et al. 2010). Their activity period is reported to be from October to April with a peak in January (Hågvar et al. 2010). During our field trips, we performed hand collection of the species around stone runs. We never observed *C. araneoides* at temperatures above freezing point. Further, we did most of our catches on windless cloudy or foggy days, but never in sunny or windy conditions.

Independent of those conditions, we caught many specimens in our traps under snow in slopes. Even under very thick (> 50 cm) and completely closed snow cover, e.g. in the cases of Lusen or Ochsenkopf (Fichtelgebirge). However, we were not able to observe any snow flies during our few field trips throughout the winter period at the snow surface of our trap sites. Nevertheless, we caught dozens up to more than hundred specimens in the traps under the surface. This suggests, that *C. araneoides* appears only temporarily on the snow surface, but generally appears to live below the snow. This is supported by the hypothesis that they need soil for reproduction (Hågvar et al. 2010). Under snow, we found living individuals only on moss cushions between rocks, where we also detected larvae of *Chionea araneoides.* Given that in most stone runs no or little substrate for larval development is found, moss and smaller substrate accumulations up to the transition to soil formations seem to be the main larval habitat. This, together with the wet, cold and constant conditions at the base of stone runs and the lack of light under the snow may be an indication for an at least facultative cave-living species. This hypothesis is further supported by the finding, that several species of the genus are known for temporarily living in caves (Avesani et al. 2016; Blick and Zaenker 2016; Oosterbroek and Reusch 2008), e.g. *C. austriaca* Christian 1980 (Novak et al. 2007) or *C. alpina* Bezzi 1908 (Vanin and Masutti 2008). Even *C. araneoides* was reported from caves (Blick and Zaenker 2016; Christian 2009). Hence, our observations indicate, that *C. araneoides* can be considered a sub-nivean rather than a supra-nivean species.

Another interesting finding, which has not been reported yet, is the existence of two different phenotypes in males: we found on the one hand large males, with a body size similar to most other *Chionea* species; on the other hand, very small males, which just reached half of the body size of the large males appeared in the traps. Such differences were not observed in females. However, we found both types of males mating with females. So far there is no clear interpretation of this finding, but two different male phenotypes may point to a complex mating system potentially with “sneaky males” (weak males), as for example also known in scarab beetles (Rasmussen 1994) and bees (Villalobos and Shelly 1991). Future behavioral observations and experiments need to confirm this.

### Sympatric records and identification problems in *C. belgica*

Different mating systems may also support the co-existence of several congeneric species. We were able to find several species of *Chionea* sympatrically at several locations. Identifications based on the external morphology indicated only two species, *C. araneoides* and *C. lutescens*, in our samples. However, DNA barcoding was able to identify the third species, *C. belgica*. Our morphological identifications were hampered by the presence/absence of a medial comb of fine bristles on sternite 9 in males, which we found also in genetically determined *C. belgica*, contradicting the key of Oosterbroek and Reusch (2008). The plasticity of this trait seemed to be high in *C. belgica*. Hence, we could not discriminate *C. belgica* from *C. lutescens* without preparation of male genitalia. Accordingly, it is likely, that former records of both species in several databases may be subject to similar misidentifications and that only barcoding may provide security on identifications.

## Conclusion

This study gives a current overview of the distribution of *Chionea araneoides* in Germany. Our records represent the first sights for federal states, i.e. Thuringia, Saxony, Saxony-Anhalt and Lower Saxony next to confirmed records for Bavaria and Hesse. The phylogenetic analysis of COI showed two clearly distinct lineages in the Bavarian forest and all other sampling sites, which probably can be explained by different refuges and migration routes during glacial periods. Ecologically *C. aranoides* shows traits of typical cave species and winter-active species. Further, we found two morphologically completely different types of males: one type (normal type) equal sized to females and other species of the genus and a second type which reaches less than half of the body size of females and “normal” males. Overall, there is much more research required to find out, how the biogeography of different lineages and populations in Germany looks like, which mating strategies may be realized in the different male types and in which unknown refugia they further can be found.
